# Diagnostic Value of the PEI-Q and Pancreatic Ultrasound for Identifying Reduced Pancreatic Exocrine Function in Early Type 2 Diabetes

**DOI:** 10.3390/jcm15208157

**Published:** 2026-10-20

**Authors:** Yerlan Bazargaliyev, Talgar Abilov, Mengtay Makashova, Khatimya Kudabayeva, Lorina Vudu

**Affiliations:** 1 Department of Internal Diseases No 1, West Kazakhstan Marat Ospanov Medical University, Aktobe 030000, Kazakhstan; yerlanbazargaliyev@gmail.com (Y.B.); hatimakudabayeva@gmail.com (K.K.); 2 Administrative and Management Personnel (AMP), West Kazakhstan Marat Ospanov Medical University, Aktobe 030000, Kazakhstan; abilovtalgar@gmail.com; 3 Department of Endocrinology, Nicolae Testemitanu State University of Medicine and Pharmacy, MD-2012 Chisinau, Moldova; lorina.vudu@usmf.md

**Keywords:** type 2 diabetes mellitus, fecal elastase-1, pancreatic exocrine function, PEI-Q, pancreatic ultrasound, Kazakhstan

## Abstract

**Background/Objectives:** Pancreatic symptoms and structural abnormalities do not necessarily correspond to reduced exocrine function in type 2 diabetes mellitus (T2DM). We aimed to estimate the frequency of reduced fecal elastase-1 (FE-1) and examine its associations with gastrointestinal symptom burden and pancreatic ultrasound findings. **Methods:** This cross-sectional study included 251 Kazakh adults with T2DM of ≤5 years’ duration not receiving insulin therapy. FE-1 < 200 μg/g was classified as reduced. Symptoms were assessed using the Pancreatic Exocrine Insufficiency Questionnaire (PEI-Q). Model 1 included pancreatic dimensions, splenic vein diameter, and Wirsung duct dilatation; Model 2 included splenic vein diameter, duct dilatation, and PEI-Q score; Model 3 additionally adjusted for age, sex, BMI, and current smoking. Discrimination was assessed using ROC analysis, with paired DeLong comparisons and Holm adjustment. **Results:** Reduced FE-1 was identified in 59 participants (23.5%) and was associated with higher PEI-Q scores, smaller pancreatic dimensions, and more frequent Wirsung duct dilatation. In Model 3, duct dilatation (adjusted OR 5.388, 95% CI 2.346–12.376) and PEI-Q score (adjusted OR 2.089, 95% CI 1.043–4.187) were independently associated with reduced FE-1. PEI-Q alone showed limited discrimination (AUC 0.583); AUCs for Models 1, 2, and 3 were 0.771, 0.697, and 0.726, respectively. Model 2 did not significantly outperform Model 1, and Model 3 did not significantly outperform Model 2. **Conclusions:** Reduced FE-1 was identified in 23.5% of participants and was associated with symptom burden and selected ultrasound findings. PEI-Q alone showed limited discrimination, whereas models incorporating ultrasound variables showed higher AUCs. Neither PEI-Q nor pancreatic ultrasound can replace FE-1 measurement; external validation is required before clinical application.

## 1. Introduction

The endocrine and exocrine compartments of the pancreas are anatomically and functionally interconnected through a shared microcirculation and bidirectional paracrine signalling across the islet–acinar axis [1]. In type 2 diabetes mellitus (T2DM), this relationship may be disrupted through several converging mechanisms, including the effects of chronic hyperglycaemia and lipotoxicity on acinar cell metabolism, pancreatic microangiopathy, autonomic dysfunction affecting secretory regulation, and low-grade inflammation and fibrosis involving the pancreatic parenchyma. These mechanisms provide a biological basis for exocrine pancreatic involvement in patients with T2DM, even in the absence of a recognized primary exocrine pancreatic disease. As such involvement may remain clinically inapparent, its detection largely relies on objective markers of pancreatic exocrine function.

Fecal elastase-1 (FE-1) is widely used for this purpose as a non-invasive marker of pancreatic exocrine function because it is stable during intestinal transit and is not affected by concurrent enzyme replacement therapy. However, a reduced FE-1 concentration alone does not fully characterize the clinical or structural manifestations of pancreatic involvement. Gastrointestinal symptoms may be absent or non-specific despite reduced FE-1, partly because of the substantial functional reserve of the pancreas. Conversely, structural pancreatic abnormalities may occur without a corresponding reduction in FE-1 or a characteristic symptom profile. A comprehensive assessment may therefore require the combined evaluation of pancreatic function, symptom burden, and structural findings.

The Pancreatic Exocrine Insufficiency Questionnaire (PEI-Q) provides a standardized patient-reported assessment of abdominal and bowel symptoms potentially associated with impaired pancreatic exocrine function. Although the PEI-Q symptom score has been validated as an outcome measure in patients with confirmed pancreatic exocrine insufficiency, evidence regarding its relationship with objectively reduced FE-1 in patients with diabetes remains limited. Parihar et al. found that PEI-Q symptom scores did not distinguish between participants with preserved and reduced FE-1, although reduced FE-1 was identified in a proportion of their study cohort [2]. Similar findings have been reported in other studies evaluating gastrointestinal symptom burden alongside FE-1 in patients with diabetes [3]. These observations suggest that symptom assessment and pancreatic function testing capture related but non-identical aspects of pancreatic exocrine involvement and that symptom burden alone may be insufficient to identify patients with reduced FE-1.

Structural assessment may provide additional information. Transabdominal ultrasonography is a routinely available, non-invasive method for evaluating pancreatic dimensions, contour, echostructure, echogenicity, and ductal abnormalities. Several studies have described pancreatic ultrasound changes in patients with T2DM. Agabi and Akhigbe reported smaller anteroposterior dimensions of the pancreatic head, body, and tail in patients with diabetes than in non-diabetic controls [4]. Irregularity and dilatation of the main pancreatic duct and its side branches have also been reported across diabetes subtypes, although these abnormalities may be subtle or absent during the early stages of disease [5]. However, individual ultrasound features have generally been examined separately, and structural imaging has rarely been evaluated directly in relation to FE-1 in patients with diabetes [6].

Consequently, the extent to which symptom-based and structural assessments provide independent or complementary information regarding reduced FE-1 remains unclear. Evidence on the joint assessment of PEI-Q symptom burden, pancreatic ultrasound findings, and FE-1 within the same cohort of patients with early T2DM remains limited. Addressing this gap may help determine whether readily available questionnaire and ultrasound findings can support a more targeted, non-invasive assessment of pancreatic exocrine function in a population in which exocrine involvement is not routinely screened for.

The present study aimed to estimate the frequency of reduced FE-1 and examine its associations with gastrointestinal symptom burden and individual pancreatic ultrasound findings.

## 2. Materials and Methods

### 2.1. Study Design and Participants

This cross-sectional observational study included adults of Kazakh nationality with T2DM of ≤5 years’ duration who were receiving outpatient endocrinology care in western Kazakhstan. Participants were recruited from outpatient clinics across western Kazakhstan between July 2025 and March 2026.

The target sample size was estimated using Epi Info based on an expected proportion of pancreatic exocrine insufficiency of 22% among individuals with T2DM, as reported in a previous systematic review and meta-analysis [7], with a 95% confidence level and an absolute precision of approximately 5%.

**Figure 1:**
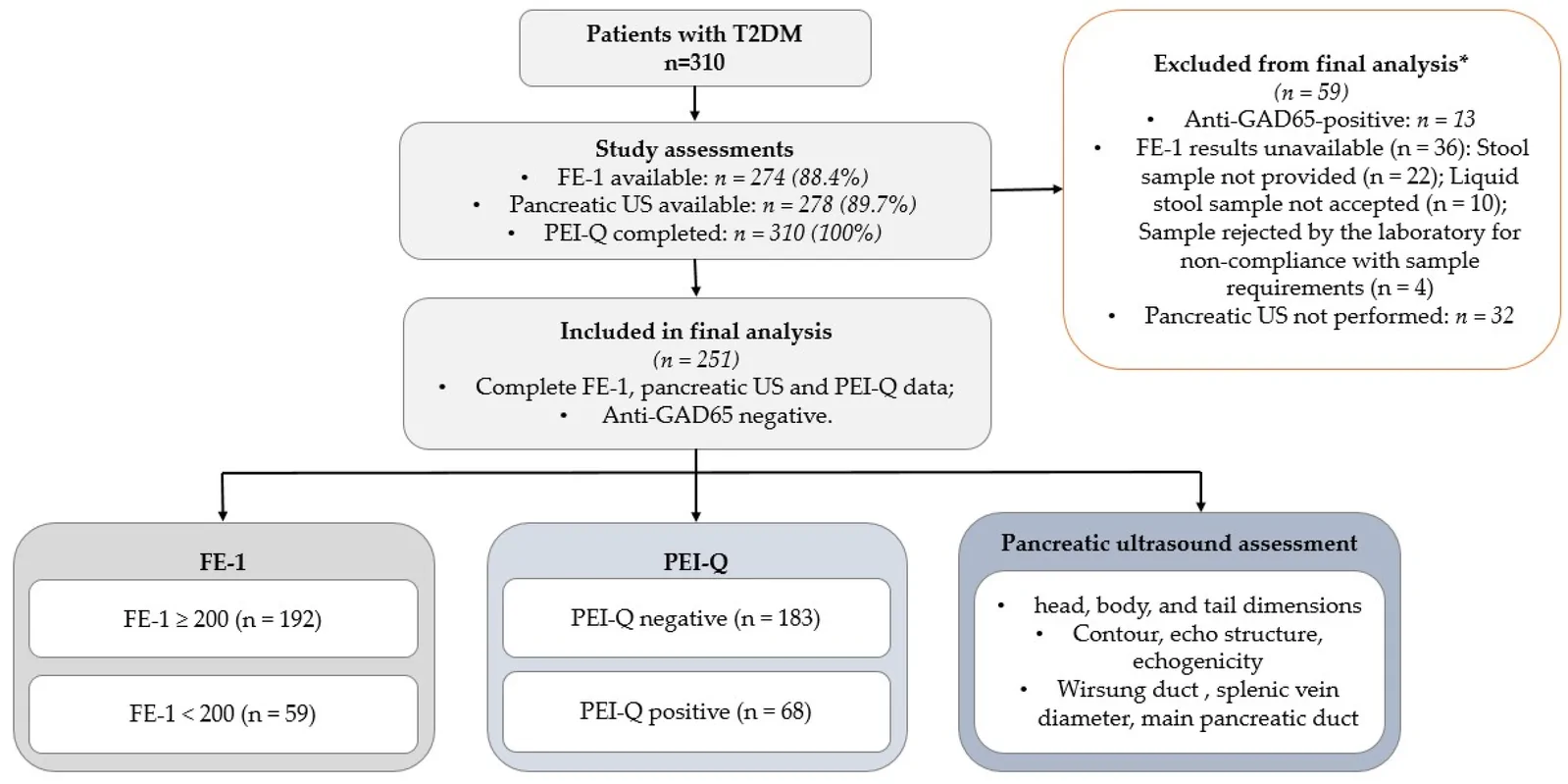
Participant flow, data availability, and analytical classification. * Reasons for exclusion were not mutually exclusive; some participants met more than one exclusion criterion.

A total of 310 participants were assessed for eligibility. FE-1 results were available for 274 participants, pancreatic ultrasound data for 278, and PEI-Q data for all 310 participants. Participants with positive anti-glutamic acid decarboxylase 65 antibodies (anti-GAD65) or incomplete data required for the predefined analyses were excluded. The final complete-case analysis included 251 participants with available FE-1, pancreatic ultrasound, and PEI-Q data and negative anti-GAD65 results. Participant selection and data availability are shown in Figure 1.

Eligible participants were adults aged 18–74 years with T2DM diagnosed according to American Diabetes Association (ADA) criteria and documented in their medical records, a diabetes duration of ≤5 years, and no episode of diabetic ketoacidosis during the preceding 6 months. Exclusion criteria were positive anti-GAD65, type 1 diabetes or another specific type of diabetes, current insulin therapy, pregnancy or lactation, malignancy, an acute or chronic infection affecting metabolic status during the preceding 4 weeks, severe decompensated chronic disease, a condition associated with systemic fibrosis, or refusal to participate.

### 2.2. Clinical and Anthropometric Assessment

Clinical data were collected using a standardized case report form and included age, sex, diabetes duration, family history of diabetes, smoking status, alcohol use, and history of gallstone disease. Body weight, height, waist circumference, and hip circumference were measured using standardized procedures. BMI was calculated as weight (kg)/height (m)^2^, and WHR as waist circumference (cm)/hip circumference (cm).

### 2.3. Fecal Elastase-1 Assessment

Exocrine pancreatic function was assessed by measuring FE-1 in a single stool sample. Participants received verbal and written instructions for sample collection. No standardized diet or specific dietary fat intake was prescribed before stool collection; participants followed their usual diet. Samples were collected without the use of enemas or laxatives, placed in sterile containers, stored at 2–8 °C until transportation, and transported under refrigerated conditions. Only solid or formed stool specimens were accepted for FE-1 measurement; semi-liquid, liquid, and watery specimens were excluded to avoid dilution-related falsely low FE-1 concentrations. FE-1 was measured by enzyme-linked immunosorbent assay using the ScheBo Pancreatic Elastase 1 Stool Test (ScheBo Biotech AG, Giessen, Germany) at the INVIVO laboratory. For the primary analyses, FE-1 concentrations < 200 μg/g were classified as reduced and concentrations ≥ 200 μg/g as preserved [8,9]. The <200 μg/g cut-off was selected to capture the broader range of reduced FE-1 concentrations rather than only values < 100 μg/g. This classification denotes reduced FE-1 and does not imply a confirmed diagnosis of pancreatic exocrine insufficiency. FE-1 concentrations were additionally categorized as <100, 100–199, and ≥200 μg/g to report their distribution and compare participant characteristics across these groups [10].

### 2.4. Pancreatic Ultrasound Assessment

Pancreatic ultrasound was performed after an overnight fast according to a standard examination protocol using a Philips Affiniti 70 ultrasound system (Philips Healthcare, Best, The Netherlands). All examinations were conducted by a single experienced physician with the highest professional qualification category who was unaware of the participants’ FE-1 and PEI-Q results. The anteroposterior dimensions of the pancreatic head, body, and tail, pancreatic contour, echostructure, echogenicity, Wirsung duct status, and splenic vein diameter were documented using a standardized ultrasound reporting form. Each participant underwent a single ultrasound examination; inter- and intraobserver agreement were not assessed.

### 2.5. Assessment of Symptoms of Pancreatic Exocrine Insufficiency

Symptoms characteristic of pancreatic exocrine insufficiency were assessed using the validated Russian-language version of the Pancreatic Exocrine Insufficiency Questionnaire (PEI-Q) (https://acarepro.abbott.com/tools/pei-q/ accessed on 14 October 2026), which was translated and linguistically validated by TransPerfect Translations International, Inc. Abbott is the copyright holder of the questionnaire, its scoring algorithm, and its translated versions. At the time of the study, preparation and linguistic validation of the Kazakh-language version were still in progress. All participants were fluent in Russian and able to understand the questionnaire.

Participants completed the questionnaire independently, based on symptoms experienced during the preceding 7 days. The overall symptom score was calculated using responses to 13 items grouped into two domains: abdominal symptoms and bowel symptoms. Each domain score was calculated as the mean of the responses to the items included in that domain. The mean overall symptom score was then calculated using the following formula: Total symptom score = (A + B)/2, where A represents the mean abdominal symptom domain score and B represents the mean bowel symptom domain score.

A mean overall symptom score of <0.60 was considered a negative PEI-Q result, whereas a score of ≥0.60 was considered positive, indicating the presence of symptoms potentially associated with pancreatic exocrine insufficiency.

### 2.6. Anti-GAD65 Antibody Testing

Anti-GAD65 antibodies were assessed using a qualitative enzyme-linked immunosorbent assay, the Human Anti-GAD65 ELISA Kit (AFG Bioscience, Arlington Heights, IL, USA; Cat. No. EK790002), in accordance with the manufacturer’s instructions https://www.afgsci.com/shop/ek790002-60358. Optical density (OD) was measured at 450 nm using a microtiter plate reader. The assay was considered valid when the mean optical density of the positive control was ≥1.00 and the mean optical density of the negative control was ≤0.10. The cut-off value was calculated using the following formula:

Cut-off value = mean optical density of the negative control + 0.15.

Samples with optical density values below the cut-off were classified as negative, whereas samples with values equal to or greater than the cut-off were classified as positive.

### 2.7. Statistical Analysis

Statistical analyses were performed using IBM SPSS Statistics for Windows, version 26.0 (IBM Corp., Armonk, NY, USA), and R, version 4.4.2 (R Foundation for Statistical Computing, Vienna, Austria). Continuous variables were assessed for normality using the Shapiro–Wilk test and are presented as median [Q1-Q3]; categorical variables are presented as *n* (%). Participants with FE-1 ≥ 200 μg/g and <200 μg/g were compared using the Mann–Whitney U test for continuous variables and Pearson’s chi-square or Fisher’s exact test for categorical variables, as appropriate. Characteristics across the three FE-1 categories (<100, 100–199, and ≥200 μg/g) were compared using the Kruskal–Wallis test and Pearson’s chi-square or Fisher’s exact test, as appropriate.

Univariable binary logistic regression analyses were performed to identify factors associated with FE-1 < 200 μg/g, reported as odds ratios (ORs) with 95% CIs. Three multivariable binary logistic regression models were then constructed. Model 1 included the pancreatic ultrasound variables (anteroposterior dimensions of the pancreatic head, body, and tail, splenic vein diameter, and Wirsung duct dilatation). Model 2 evaluated whether the PEI-Q total symptom score added explanatory value beyond the venous and ductal variables retained in Model 1 (splenic vein diameter and Wirsung duct dilatation), as the pancreatic dimension variables were not independently associated with reduced FE-1. Model 3 additionally adjusted for age, sex, BMI, and current smoking. Covariate selection combined univariable findings with clinical considerations: sex and current smoking were included based on their significant univariable associations, whereas age and BMI were retained as clinically relevant adjustment variables irrespective of statistical significance [11,12]. Results are reported as adjusted ORs with 95% CIs. Model fit was assessed using the omnibus likelihood-ratio chi-square test, Nagelkerke R^2^, and the Hosmer–Lemeshow test. Linearity in the logit for continuous predictors was assessed using likelihood-ratio tests comparing linear effects with natural cubic spline specifications with three degrees of freedom, with Holm adjustment within each model. Multicollinearity was assessed using variance inflation factors (VIFs), and the sensitivity of the model estimates to individual observations was explored using individual case-deletion analyses.

ROC analysis evaluated the ability of the PEI-Q total symptom score and Models 1–3 to identify FE-1 < 200 μg/g. Results are reported as AUCs with 95% CIs, and paired DeLong comparisons with Holm adjustment were used to compare AUCs between models. All tests were two-sided, with *p* < 0.05 considered statistically significant.

## 3. Results

### 3.1. Study Population and FE-1 Status

A total of 251 patients with type 2 diabetes mellitus were included in the analysis. The median age was 56 [47.0–62.0] years. Women predominated in the cohort, accounting for 175 patients (69.7%), whereas 76 patients (30.3%) were men. The median BMI was 30.5 [27.2–34.0] kg/m^2^, and the median waist-to-hip ratio was 0.96 [0.92–1.02]. Current smoking was reported by 33 patients (13.1%), and 68 patients (27.1%) had a history of gallstone disease.

FE-1 was used as a marker of exocrine pancreatic function. The median FE-1 level in the overall cohort was 480.06 [219.6–500.0] μg/g. Using the prespecified threshold of 200 μg/g, 59 patients (23.5%) had reduced FE-1, including 39 (15.5%) with concentrations of 100–199 μg/g and 20 (8.0%) with concentrations < 100 μg/g. The remaining 192 patients (76.5%) had FE-1 ≥ 200 μg/g.

### 3.2. Clinical and Demographic Characteristics According to FE-1 Status

To identify clinical and demographic characteristics associated with FE-1 levels, the evaluated variables were compared across three FE-1 categories (Table 1). Current smoking was more frequent in the FE-1 100–199 μg/g and <100 μg/g groups than in the FE-1 ≥ 200 μg/g group (23.1% and 25.0% vs. 9.9%, respectively; *p* = 0.022). No other significant differences in the evaluated clinical and demographic characteristics were observed across the three groups.

### 3.3. Pancreatic Ultrasound Findings According to FE-1 Status

Pancreatic ultrasound findings were subsequently compared between the FE-1-defined groups (Table 2). Patients with FE-1 < 200 μg/g had smaller pancreatic head (21.0 [18.0–26.6] vs. 24.0 [20.25–29.0] mm; *p* = 0.005), body (13.0 [11.0–16.0] vs. 15.0 [12.33–17.0] mm; *p* = 0.010), and tail dimensions (19.0 [16.0–21.0] vs. 20.0 [18.0–22.83] mm; *p* = 0.002). The splenic vein diameter was larger in patients with FE-1 < 200 μg/g than in those with FE-1 ≥ 200 μg/g (8.0 [5.0–9.0] vs. 6.0 [5.0–8.0] mm; *p* = 0.028), and Wirsung duct dilatation was more frequent (28.8% vs. 6.8%; *p* < 0.001). No significant between-group differences were observed in pancreatic contour, echostructure, or echogenicity.

### 3.4. PEI-Q Symptom Burden According to FE-1 Status

Having identified ultrasound differences between the FE-1-defined groups, we next examined whether FE-1 < 200 μg/g was also associated with a greater symptom burden, as assessed using the PEI-Q. The total PEI-Q symptom score was significantly higher in patients with FE-1 < 200 μg/g than in those with FE-1 ≥ 200 μg/g (0.286 [0.07–0.92] vs. 0.071 [0.07–0.52], respectively; *p* = 0.049). PEI-Q positivity, defined as a total symptom score ≥ 0.60, was also more frequent in patients with FE-1 < 200 μg/g than in those with FE-1 ≥ 200 μg/g (35.6% vs. 24.5%); however, this difference did not reach statistical significance (*p* = 0.093) (Table 3).

### 3.5. Associations of Symptom-Based and Ultrasound Parameters with Reduced FE-1

To evaluate whether symptom burden and pancreatic ultrasound parameters were associated with reduced exocrine pancreatic function, binary logistic regression analyses were performed using FE-1 < 200 μg/g as the dependent outcome. In univariable analyses, higher PEI-Q total symptom score, male sex, current smoking, greater splenic vein diameter, and main pancreatic duct dilation were associated with higher odds of reduced FE-1, whereas larger pancreatic head, body, and tail sizes were associated with lower odds. Age, BMI, alcohol consumption, history of gallstone disease, irregular pancreatic contour, heterogeneous pancreatic structure, and altered pancreatic echogenicity were not significantly associated with reduced FE-1 (Table 4).

**Figure 2:**
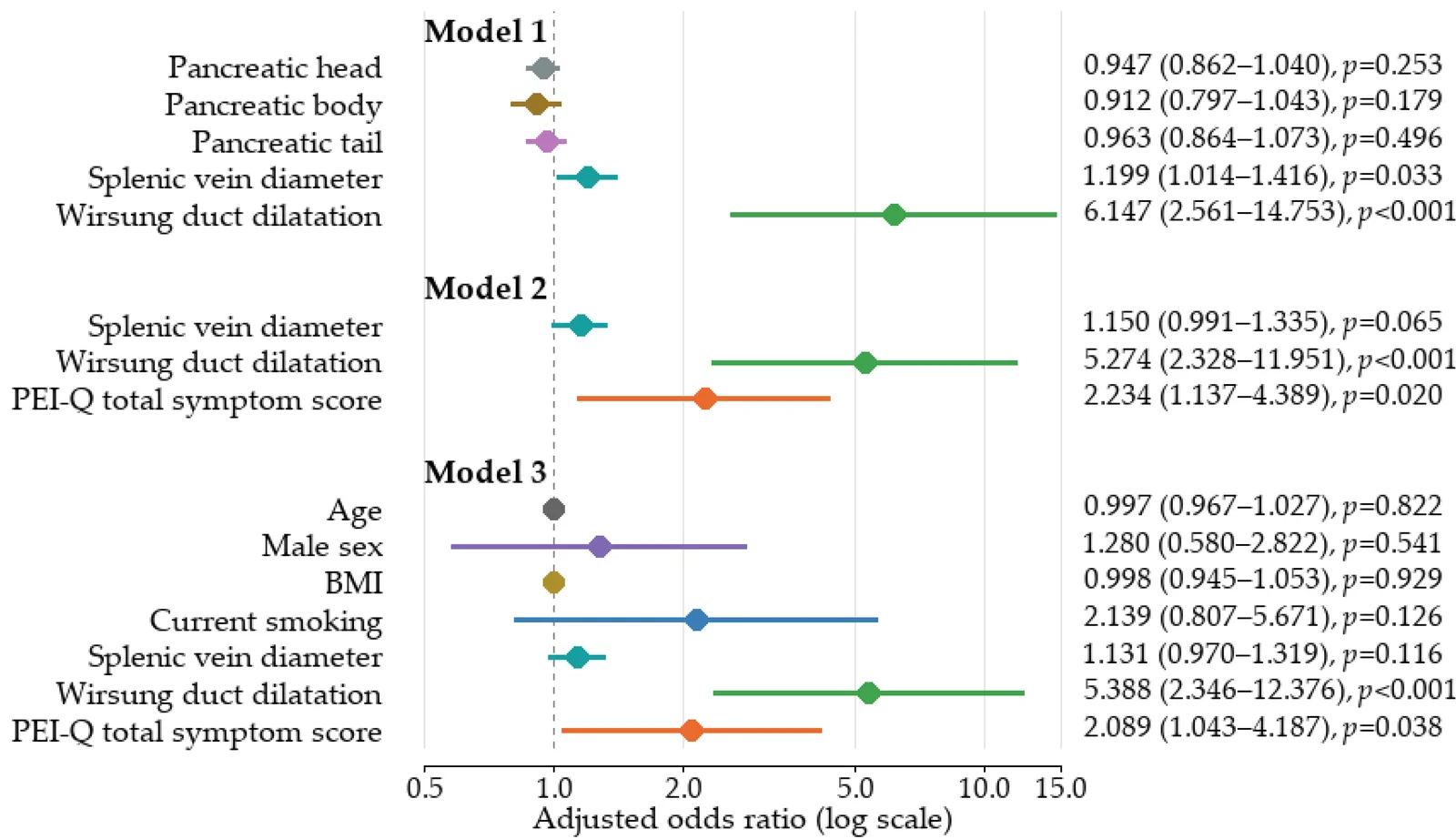
Forest plot of adjusted odds ratios for factors associated with FE-1 < 200 μg/g across the three multivariable logistic regression models (Model 1: pancreatic ultrasound variables; Model 2: splenic vein diameter, Wirsung duct dilatation, and the PEI-Q total symptom score; Model 3: fully adjusted for age, sex, BMI, and current smoking).

To further examine whether pancreatic ultrasound characteristics and symptom burden were independently associated with reduced FE-1, three multivariable logistic regression models were constructed (Figure 2). In Model 1, larger splenic vein diameter and Wirsung duct dilatation were associated with FE-1 < 200 μg/g, whereas pancreatic head, body, and tail dimensions were not. When the PEI-Q total symptom score was included alongside splenic vein diameter and Wirsung duct dilatation in Model 2, both duct dilatation and a higher symptom score were significantly associated with reduced FE-1, whereas splenic vein diameter was no longer statistically significant. In Model 3, Wirsung duct dilatation and a higher PEI-Q total symptom score remained independently associated with FE-1 < 200 μg/g, whereas age, sex, BMI, current smoking, and splenic vein diameter were not. The final model was statistically significant overall (Omnibus χ^2^ = 31.352, df = 7, *p* < 0.001; Nagelkerke R^2^ = 0.177), with no evidence of lack of fit according to the Hosmer–Lemeshow test (*p* = 0.144).

No significant departures from linearity were detected after Holm adjustment, and VIF values ranged from 1.01 to 2.35. Individual case-deletion analyses indicated that the statistical significance of the splenic vein association in Models 1–2 and the PEI-Q association in Model 3 was sensitive to certain observations, although their directions were preserved. The Wirsung duct dilatation association retained its direction and statistical significance. All participants were retained in the primary analyses. Detailed diagnostics are presented in Supplementary Table S1.

### 3.6. Diagnostic Value of PEI-Q and Pancreatic Ultrasound for Identifying Reduced FE-1

**Figure 3:**
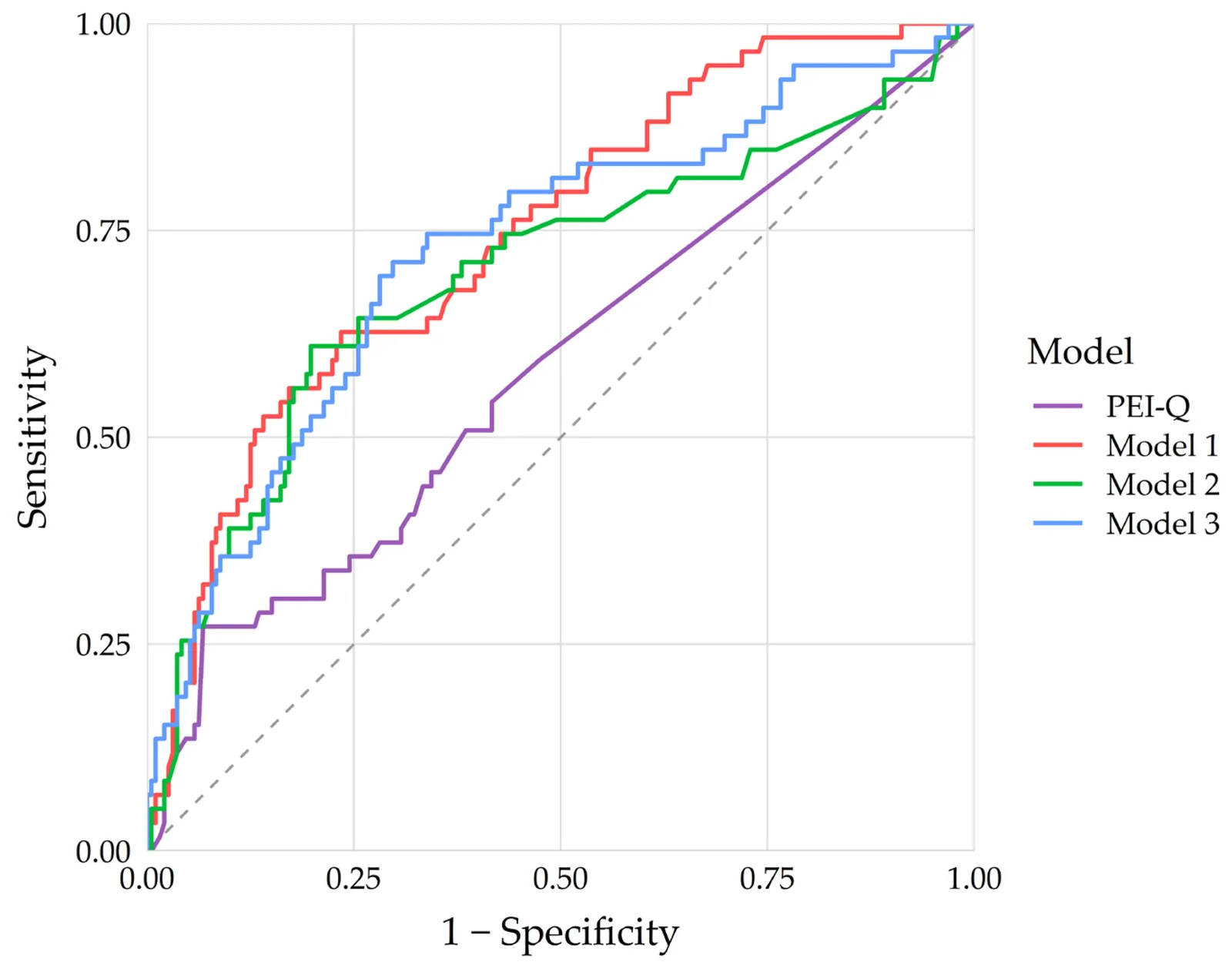
Receiver operating characteristic curves for the PEI-Q total symptom score alone, ultrasound-based model (Model 1), combined symptom–ultrasound model (Model 2), and clinically adjusted model (Model 3) for identifying FE-1 < 200 μg/g. The dashed diagonal line represents chance discrimination (AUC = 0.5).

ROC analysis was performed to compare the discriminatory performance of the PEI-Q total symptom score and Models 1–3 for identifying participants with FE-1 < 200 μg/g. The ultrasound-based Model 1 showed the numerically highest AUC (0.771, 95% CI 0.705–0.838), followed by the clinically adjusted Model 3 (0.726, 95% CI 0.649–0.803) and the combined symptom–ultrasound Model 2 (0.697, 95% CI 0.612–0.782). PEI-Q alone showed limited discrimination (AUC 0.583, 95% CI 0.496–0.670). In paired DeLong comparisons, Model 2 had a higher AUC than PEI-Q (ΔAUC = 0.115, 95% CI 0.022–0.207; Holm-adjusted *p* = 0.046), whereas differences between Models 1 and 2 and between Models 3 and 2 were not statistically significant (Holm-adjusted *p* = 0.187 and 0.268, respectively) (Table 5 and Figure 3). Detailed results of the pairwise AUC comparisons are presented in Supplementary Table S2.

## 4. Discussion

In this cross-sectional study of 251 Kazakh adults with T2DM of ≤5 years’ documented duration, reduced FE-1 (<200 μg/g) was identified in 23.5% of participants. This frequency was close to the pooled prevalence of approximately 22% reported by Zhang et al. [7], although estimates in previous studies of patients with T2DM have ranged from approximately 17% to 49% [13]. Parihar et al. reported reduced FE-1 in 12% of a mixed cohort of patients with diabetes using the same threshold [2]. These differences may reflect variation in study populations and eligibility criteria. Our cohort included patients who were not receiving insulin, and anti-GAD65-positive participants were excluded. The findings suggest that reduced FE-1 may already be present within the first years after the documented diagnosis of T2DM. FE-1 concentrations were 100–199 μg/g in 15.5% of participants and <100 μg/g in 8.0%. These results describe the frequency of reduced FE-1, which alone does not establish a diagnosis of pancreatic exocrine insufficiency [10].

Current smoking and male sex were associated with reduced FE-1 in univariable analyses, but neither association remained statistically significant in Model 3. The smoking association is consistent with findings by Parihar et al. in patients with diabetes [2] and de Wit et al. in individuals with type 1 diabetes [14], although Icks et al. reported no association with current or lifetime smoking [15]. Experimental evidence suggests that nicotine exposure may disturb pancreatic acinar cell function and promote oxidative stress, providing a possible biological explanation for an association between smoking and pancreatic exocrine dysfunction [16]. The attenuation of the smoking and sex associations after adjustment suggests that they may reflect shared exposures or other participant characteristics. Differences in smoking exposure or body composition may contribute to the observed sex association, although these explanations were not examined in this study.

Age and BMI were not independently associated with reduced FE-1. Nevertheless, they were retained in Model 3 as clinically relevant adjustment variables, irrespective of statistical significance. Previous studies have reported inconsistent associations between these clinical factors and exocrine pancreatic function: Ewald et al. reported an association between FE-1 concentrations and BMI, but no association with age [11], whereas Yilmaztepe et al. and Terzin et al. found no significant correlations of FE-1 with BMI or age [17,18]. The absence of these associations in our study may partly reflect the restriction of the cohort to patients with a diabetes duration of no more than five years and the exclusion of insulin-treated patients, resulting in a clinically homogeneous population with a relatively narrow range of disease duration.

Several ultrasound findings differed between the FE-1 groups. Patients with reduced FE-1 had smaller pancreatic head, body, and tail dimensions and more frequent Wirsung duct dilatation. The smaller pancreatic dimensions are consistent with previous imaging studies demonstrating reduced pancreatic volume in patients with diabetes; loss of pancreatic volume may reflect acinar tissue loss, fatty replacement, microvascular injury, or fibrotic remodelling [5,19,20]. These mechanisms cannot, however, be distinguished using the ultrasound measurements obtained in this study. Pancreatic dimensions were not independently associated with reduced FE-1 in Model 1 and were therefore not retained in Models 2 and 3. Wirsung duct dilatation remained independently associated with reduced FE-1 in Model 3 (adjusted OR 5.388, 95% CI 2.346–12.376), with dilatation present in 28.8% of participants with reduced FE-1 compared with 6.8% of those with preserved FE-1. Ductal abnormalities have previously been described across diabetes subtypes [5,6], and duct dilatation may indicate fibrotic or chronic inflammatory remodelling of the pancreatic parenchyma.

Splenic vein diameter was associated with reduced FE-1 in the univariable analysis and Model 1, but did not retain statistical significance in Models 2 and 3; case-deletion analyses indicated sensitivity to individual observations. This association warrants further investigation to clarify its relationship with pancreatic exocrine function. Pancreatic contour, echostructure, and echogenicity did not differ between the FE-1 groups, despite the high frequency of abnormalities in the overall cohort. These features are not specific to reduced exocrine function and may be influenced by fatty infiltration, obesity, age-related changes, and examination technique [20,21], which is relevant given the median BMI of 30.5 kg/m^2^ in our cohort. Moreover, structural and functional pancreatic changes may not develop in parallel because the pancreas has considerable functional reserve [22]; mild structural abnormalities may therefore coexist with preserved exocrine function, while functional decline may precede detectable structural changes.

The PEI-Q total symptom score was higher in participants with reduced FE-1 and was associated with reduced FE-1 in the univariable analysis and in Models 2 and 3. However, PEI-Q positivity based on the prespecified threshold of ≥0.60 did not differ significantly between the groups. Parihar et al. similarly found that a symptom-based scoring system did not clearly distinguish between patients with diabetes with reduced and preserved FE-1 [2]. These findings suggest that the continuous PEI-Q score may capture differences in overall gastrointestinal symptom burden that are not fully reflected by a single dichotomous threshold. Because gastrointestinal symptoms in patients with diabetes may have multiple causes, the PEI-Q should be interpreted as a structured measure of symptom burden rather than as direct evidence of reduced exocrine pancreatic function.

The ROC analysis showed that Model 1 had the numerically highest AUC (0.771), suggesting that selected ultrasound findings provided information relevant to identifying reduced FE-1. Model 2 showed significantly higher discrimination than PEI-Q alone but did not demonstrate significantly better discrimination than Model 1. Because Models 1 and 2 differed both in the inclusion of PEI-Q and in the ultrasound variables retained, their comparison does not isolate the incremental contribution of symptom assessment. Clinical adjustment in Model 3 did not significantly improve discrimination compared with Model 2. Thus, the evaluated combined model did not outperform the ultrasound-based model, and adding clinical covariates did not demonstrate a further discriminatory benefit. Although transabdominal ultrasound may miss subtle changes detectable by MRI/MRCP or endoscopic ultrasound [23], and pancreatic morphology does not always correspond to exocrine function [22], our findings suggest that individual ultrasound features can contribute to the assessment of pancreatic involvement alongside FE-1 measurement. The present findings do not support using these models to determine whether FE-1 testing is needed or to rule out reduced FE-1, given the risk of false-negative results.

Several limitations should also be acknowledged. The cross-sectional design precludes causal inference and does not allow assessment of whether reduced FE-1 progresses, remains stable, or resolves over time. FE-1 was measured in a single stool sample without confirmation in a second specimen, which is a general limitation of single-measurement FE-1 testing. Because only formed or solid stool specimens were analysed, the findings may not be generalizable to patients with diarrhoea. No standardized diet was prescribed before stool collection, and the potential influence of dietary variation was not assessed. Ultrasound reproducibility was not assessed; therefore, small between-group differences in pancreatic dimensions should be interpreted cautiously because measurement variability cannot be excluded. The validated Russian-language PEI-Q was used, while the Kazakh-language version was still under development; its measurement properties were not evaluated specifically in this cohort. Glycemic control and detailed glucose-lowering medication use were not accounted for in the analyses, leaving the possibility of residual confounding. The sample-size calculation was based on prevalence estimation and was not specifically designed for diagnostic performance analyses or multivariable modelling, which should therefore be considered exploratory. The models were developed and evaluated within the same cohort without internal validation. The reported AUCs therefore represent apparent discrimination and may overestimate performance in other populations; external validation is required. Finally, because eligibility was restricted to patients with a T2DM duration of five years or less who were not receiving insulin therapy, our findings cannot be extrapolated to patients with longer-standing or insulin-treated diabetes, in whom exocrine involvement may be more advanced.

## 5. Conclusions

In conclusion, reduced FE-1 was identified in 23.5% of Kazakh adults with T2DM of ≤5 years’ documented duration and was associated with higher PEI-Q total symptom scores and selected pancreatic ultrasound findings. PEI-Q alone showed limited discrimination, whereas models incorporating individual ultrasound variables showed higher discriminatory performance. However, the combined symptom–ultrasound model did not show significantly better discrimination than the ultrasound-based model. Neither PEI-Q nor pancreatic ultrasound can replace FE-1 measurement, and the models require external validation before clinical application.

## Figures and Tables

**Table 1:** Comparison of clinical and demographic characteristics across FE-1 categories.

Variable	FE-1 ≥ 200 μg/g (*n* = 192)	FE-1 100–199 μg/g (*n* = 39)	FE-1 < 100 μg/g (*n* = 20)	*p*-Value
	**Median [Q_1_–Q_3_]/** ***n*** **(%)**			
Age, years	56 [47.25–62.00]	55 [46.00–60.00]	57 [48.00–66.00]	0.487
Sex, male	52 (27.1)	16 (41.0)	8 (40.0)	0.138
BMI, kg/m^2^	30.48 [27.38–34.05]	29.41 [26.90–34.82]	30.41 [26.67–33.94]	0.879
WHR	0.96 [0.91–1.02]	0.96 [0.93–1.02]	0.98 [0.92–1.05]	0.420
Current smoking	19 (9.9)	9 (23.1)	5 (25.0)	**0.022**
Alcohol consumption	32 (16.7)	8 (20.5)	6 (30.0)	0.317
History of gallstone disease	53 (27.6)	9 (23.1)	6 (30.0)	0.807

Continuous variables were compared using the Kruskal–Wallis test; categorical variables were compared using Pearson’s chi-square test or Fisher’s exact test, as appropriate.

**Table 2:** Comparison of pancreatic ultrasound findings between FE-1 groups.

Variable	FE-1 < 200 μg/g (*n* = 59)	FE-1 ≥ 200 μg/g (*n* = 192)	*p*-Value
	Median [Q_1_–Q_3_]		
Pancreatic head size, mm	21.0 [18.0–26.6]	24.0 [20.25–29.0]	**0.005**
Pancreatic body size, mm	13.0 [11.0–16.0]	15.0 [12.33–17.0]	**0.010**
Pancreatic tail size, mm	19.0 [16.0–21.0]	20.0 [18.0–22.83]	**0.002**
Splenic vein diameter, mm	8.0 [5.0–9.0]	6.0 [5.0–8.0]	**0.028**
Pancreatic contour, *n* (%)			
Regular	31 (52.5)	75 (39.1)	**0.067**
Irregular	28 (47.5)	117 (60.9)	
Pancreatic echostructure, *n* (%)			
Homogeneous	17 (28.8)	39 (20.3)	0.170
Heterogeneous	42 (71.2)	153 (79.7)	
Pancreatic echogenicity, *n* (%)			
Normal	10 (16.9)	45 (23.4)	0.292
Altered	49 (83.1)	147 (76.6)	
Wirsung duct status, *n* (%)			
Not dilated	42 (71.2)	179 (93.2)	**<0.001**
Dilated	17 (28.8)	13 (6.8)	

Continuous variables were compared using the Mann–Whitney U test; categorical variables were compared using Pearson’s chi-square test or Fisher’s exact test, as appropriate.

**Table 3:** Comparison of PEI-Q symptom scores and positivity between FE-1 groups.

Variable	FE-1 < 200 μg/g (*n* = 59)	FE-1 ≥ 200 μg/g (*n* = 192)	*p*-Value
Total PEI-Q symptom score Median [Q_1_–Q_3_]	0.286 [0.07–0.92]	0.071 [0.07–0.52]	**0.049**
PEI-Q negative *n* (%)	38 (64.4)	145 (75.5)	0.093
PEI-Q positive *n* (%)	21 (35.6)	47 (24.5)	

Continuous variables were compared using the Mann–Whitney U test; categorical variables were compared using Pearson’s chi-square test or Fisher’s exact test, as appropriate.

**Table 4:** Univariable logistic regression analysis of factors associated with reduced FE-1 (<200 μg/g).

Predictor	β	OR (95% CI)	*p*-Value
Age, (per 1-year increase)	−0.001	0.999 (0.972–1.027)	0.968
Male sex	0.613	1.846 (1.004–3.390)	**0.049**
BMI, (per 1 kg/m^2^ increase)	0.001	1.001 (0.951–1.053)	0.971
Current smoking	1.041	2.833 (1.319–6.098)	**0.008**
Alcohol consumption	0.442	1.555 (0.765–3.165)	0.222
History of gallstone disease	0.112	1.118 (0.575–2.177)	0.742
PEI-Q total symptom score, (per 1-unit increase)	0.688	1.989 (1.044–3.789)	**0.036**
Pancreatic head size (per 1 mm increase)	−0.079	0.924 (0.875–0.977)	**0.005**
Pancreatic body size (per 1 mm increase)	−0.115	0.892 (0.815–0.976)	**0.013**
Pancreatic tail size (per 1 mm increase)	−0.119	0.888 (0.823–0.959)	**0.002**
Splenic vein diameter (per 1 mm increase)	0.172	1.187 (1.030–1.368)	**0.018**
Irregular pancreatic contour (present vs. absent)	−0.546	0.579 (0.322–1.042)	0.068
Heterogeneous pancreatic structure (present vs. absent)	−0.462	0.630 (0.324–1.223)	0.172
Altered pancreatic echogenicity (present vs. absent)	0.405	1.500 (0.703–3.200)	0.294
Dilated main pancreatic duct (present vs. absent)	1.718	5.573 (2.513–12.360)	**<0.001**

**Table 5:** Diagnostic performance of ultrasound-based and combined symptom–ultrasound models for identifying reduced FE-1.

Model	AUC	SE	95% CI
PEI-Q total symptom score	0.583	0.044	0.496–0.670
Model 1	0.771	0.034	0.705–0.838
Model 2	0.697	0.043	0.612–0.782
Model 3	0.726	0.039	0.649–0.803

## Data Availability

The data presented in this study are available on request from the corresponding author.

## Supplementary Materials

The following supporting information can be downloaded at: https://www.mdpi.com/article/10.3390/jcm15208157/s1, Table S1: Assessment of linearity, multicollinearity, and influential observations in logistic regression Models 1–3; Table S2: Pairwise comparisons of AUCs using paired DeLong tests with Holm adjustment.

## Abbreviations

ADA	American Diabetes Association
anti-GAD65	Antibodies to glutamic acid decarboxylase 65
AUC	Area under the receiver operating characteristic curve
BMI	Body mass index
CI	Confidence interval
ELISA	Enzyme-linked immunosorbent assay
FE-1	Fecal elastase-1
OR	Odds ratio
PEI-Q	Pancreatic Exocrine Insufficiency Questionnaire
ROC	Receiver operating characteristic
SE	Standard error
SPSS	Statistical Package for the Social Sciences
T2DM	Type 2 diabetes mellitus
WHR	Waist-to-hip ratio
