# Supplementary material for: Diagnostic Value of the PEI-Q and Pancreatic Ultrasound for Identifying Reduced Pancreatic Exocrine Function in Early Type 2 Diabetes
Source: J Clin Med. 2026 Oct 20;15(20):8157. doi: 10.3390/jcm15208157 (PMC13705915; doi:10.3390/jcm15208157)
Supplement: Supplementary file 1 [file jcm-15-08157-s001.zip › jcm-4542149-supplementary.pdf]

Table S1. Assessment of linearity, multicollinearity, and influential observations in logistic regression Models 1–3.

Diagnostic assessment / predictor	Model 1	Model 2	Model 3
**A. Linearity in the logit: p-value (Holm-adjusted p-value)**			
Pancreatic head size	0.872 (1.000)	-	-
Pancreatic body size	0.410 (1.000)	-	-
Pancreatic tail size	0.863 (1.000)	-	-
Splenic vein diameter	0.045 (0.179)	0.135 (0.269)	0.081 (0.325)
PEI-Q total symptom score	-	>0.999 (>0.999)	0.978 (0.978)
Age	-	-	0.143 (0.429)
BMI	-	-	0.386 (0.772)
**B. Multicollinearity: VIF**			
Pancreatic head size	2.346	-	-
Pancreatic body size	1.862	-	-
Pancreatic tail size	1.720	-	-
Splenic vein diameter	1.173	1.017	1.043
Wirsung duct dilatation	1.068	1.022	1.036
PEI-Q total symptom score	-	1.014	1.022
Age	-	-	1.034
Sex	-	-	1.455
Current smoking	-	-	1.428
BMI	-	-	1.056
**C. Influence diagnostics**			
Number of participants	251	251	251
Number of parameters, including intercept	6	4	8
Maximum Cook's distance	0.0731	0.0388	0.0406
Maximum leverage	0.1368	0.0937	0.1330
Maximum absolute DFBETA	0.5191	0.3760	0.4375
Observations with Cook's distance >4/n, count	18	22	25
Observations with leverage >2k/n, count	23	38	27
Observations with any absolute DFBETA >2/√n, count	73	64	86
Observations exceeding ≥1 screening threshold, count	77	64	86
Note: Linearity was assessed using likelihood-ratio tests comparing linear effects with natural cubic spline specifications with three degrees of freedom. Holm adjustment was applied within each model; adjusted p-values are shown in parentheses in section A. VIF, variance inflation factor; BMI, body mass index; n, sample size; k, number of model parameters including the intercept. A			

dash indicates that the predictor was not included in the model. Screening thresholds were not used as automatic exclusion criteria.
Individual case-deletion analyses indicated that the statistical significance of the splenic vein association in Models 1 and 2 and the PEI-Q association in Model 3 was sensitive to certain observations, while their directions were preserved. The Wirsung duct dilatation association retained its direction and statistical significance in these analyses. All 251 participants were retained in the primary analyses.

Table S2. Pairwise comparisons of AUCs using paired DeLong tests with Holm adjustment.

Comparison	ΔAUC	95% CI for ΔAUC	p-value	Holm-adjusted p-value
Model 2 vs. PEI-Q	0.115	0.022–0.207	0.015	**0.046**
Model 2 vs. Model 1	−0.074	−0.160–0.013	0.094	0.187
Model 3 vs. Model 2	0.029	−0.022–0.080	0.268	0.268
